# Minimum Conflict Individual Haplotyping from SNP Fragments and Related Genotype

**Published:** 2007-02-16

**Authors:** Xiang-Sun Zhang, Rui-Sheng Wang, Ling-Yun Wu, Wei Zhang

**Affiliations:** 1 Academy of Mathematics and Systems Science, Chinese Academy of Sciences, Beijing 100080, China; 2 School of Information, Renmin University of China, Beijing 100872; 3 North Carolina State University, Raleigh, NC 27695-7906, U.S.A

**Keywords:** individual haplotyping, minimum conflict individual haplotyping, NP-hard, dynamic programming, feed-forward neural network, reconstruction rate

## Abstract

The Minimum Error Correction (MEC) is an important model for haplotype reconstruction from SNP fragments. However, this model is effective only when the error rate of SNP fragments is low. In this paper, we propose a new computational model called Minimum Conflict Individual Haplotyping (MCIH) as an extension to MEC. In contrast to the conventional approaches, the new model employs SNP fragment information and also related genotype information, thereby a high accurate inference can be expected. We first prove the MCIH problem to be NP-hard. To evaluate the practicality of the new model we design an exact algorithm (a dynamic programming procedure) to implement MCIH on a special data structure. The numerical experience indicates that it is fairly effective to use MCIH at the cost of related genotype information, especially in the case of SNP fragments with a high error rate. Moreover, we present a feed-forward neural network algorithm to solve MCIH for general data structure and large size instances. Numerical results on real biological data and simulation data show that the algorithm works well and MCIH is a potential alternative in individual haplotyping.

## Introduction

The availability of complete genome sequence for human beings [Vent et al. 2001] makes it possible to investigate genetic differences and to associate genetic variations with complex diseases [Hoehe et al. 2000]. Single nucleotide polymorphism (SNP)—a single DNA base varying from one individual to another, is believed to be the most frequent form to address genetic differences [Chakravarti, 1998; Li et al. 2005]. SNPs are found approximately every 1000 base pairs in the human genome and turn to be promising tools for doing disease association study. Many research works have been carried out for determining SNP sites or designing a detailed SNP map for human genome [Altshuler et al. 2000; Helmuth, 2001].

The nucleotides in a SNP position are called *alleles*. Almost all SNPs have two different alleles which we denote the wild type as 1 and the mutant type as −1. The SNP sequence information on each copy of a pair of chromosomes in a diploid genome is called a *haplotype* which is a string over {−1, 1}. A *genotype* is the conflated information of a pair of haplotypes on homologous chromosomes. For a genotype, if a pair of alleles at a SNP site is made of two identical values, this SNP site is called *homozygous*, otherwise it is called *heterozygous*.

Haplotypes generally have more information content than that individual SNPs have in disease association studies [Stephens et al. 2001], but it is substantially more difficult to determine haplotypes than to determine genotypes or individual SNPs through experiments. Hence, computational methods that can reduce the cost of determining haplotypes become attractive alternatives. There are generally two classes of computational methods for determining haplotype. One class concerns with infering haplotypes from the genotype samples in a population. There are several models based on different assumptions on the biological system under consideration [Clark, 1990; Gusfield, 2002; Halperin and Eskin, 2004; Li et al. 2005; Wang and Xu, 2003]. The second class, called single individual haplotyping or haplotype assembly, is based on the data and methodology of shotgun sequence assembly [Lancia et al. 2001; Lippert et al. 2002]. The input data consists of aligned short genome fragments with SNPs coming from DNA shotgun sequencing or data generated by a resequencing effort for the purpose of large-scale haplotyping. When we focus on SNP positions, these short genome fragments are actually the aligned SNP fragments.

Computationally, the individual haplotyping problem is to determine the “best” pair of haplotypes from data (SNP fragments) which is possibly inconsistent and contradictory. It focuses on partitioning SNP fragments into two sets according to SNP states, with each set determining a haplotype [Lancia et al. 2001; Lippert et al. 2002]. For such a problem, there are several models based on different error assumptions [Lancia et al. 2001; Li, L.M. et al. 2004; Lippert et al. 2002; Rizzi et al. 2002; Wang et al. 2005], of which the Minimum Error Correction model (MEC) is widely adopted. MEC assumes that the inconsistence of the data comes from realistic sequence errors and these errors can be corrected. However, MEC is only effective in the case that SNP fragments have a low error rate. When the error rate of SNP fragments is relatively high, we can not reconstruct original haplotypes with a high accuracy by error correction only [Wang et al. 2005]. To improve the haplotyping quality, we need to either reduce the errors in SNP fragments which will call for improvement of the shortgun experiment, or add extra information to the given SNP fragment set. Since genotype data can be much more easily (and also economically) obtained, a computational model combining both SNP fragments and genotype information will be a realistic strategy. The idea is motivated by the method in [Eskin et al. 2004]. In their paper they phased long genotypes using local haplotype information. In this paper, we propose a new computational model (Minimum Conflict Individual Haplotyping: MCIH) for individual haplotyping by using this strategy, which is also proved to be NP-hard.

There are two ways to show that a new established model is practical or more effective than an existing model: one is to theoretically prove that the solution of the new model is superior to that of the existing model, the other is to numerically solve the problem and compare the solutions obtained by the two models. It is obvious that we can only do it in the second way. For this reason we try to design an exact algorithm for the MCIH problem. A special data structure suited for a dynamic programming algorithm is displayed and used to evaluate the model. Computational results by this exact algorithm confirm the significance of MCIH. Moreover, a feed-forward neural network (FNN) is designed for computing approximate solutions to the problem (MCIH) in general case and of large size. Extensive computational results show the effectiveness of the proposed FNN algorithm and MCIH.

The paper is organized as follows: In Section I, we give the problem definition together with the complexity analysis of MCIH. The model evaluation is shown in Section II. In Section III, a feed-forward neural network algorithm is designed for implementing MCIH. Experiment results are given in Section IV. Section V concludes the paper.

### I. Formulation and Problem

For a genotype ***g*** = (*g*_1_, *g*_2_, ···, *g_n_*), if the *j*th SNP site is wild type homozygous, *g_j_* = 2; if it is mutant type homozygous, *g_j_* = −2; when it is heterozygous, *g_j_* = 0. A pair of haplotypes ***h***_1_ = (*h*_11_, *h*_12_, ···, *h*_1_*_n_*) and ***h***_2_ = (*h*_21_, *h*_22_, ···, *h*_2_*_n_*) is called *compatible* with a genotype ***g*** if the following conditions hold: for each SNP site *j* where *g_j_* = −2, *h*_1_*_j_* = *h*_2_*_j_* = −1; for each SNP site *j* where *g_j_* = 2, *h*_1_*_j_* = *h*_2_*_j_* = 1; for each SNP site *j* where *g_j_* = 0, *h*_1_*_j_* = −*h*_2_*_j_* = −1 or *h*_1_*_j_* = − *h*_2_*_j_* = 1.

Suppose that there are *m* SNP fragments from a pair of chromosomes and the length of each corresponding haplotype is *n*. Define an *m* × *n* SNP matrix ***M*** = (*m_ij_*), whose entry *m_ij_* has the value −1, 1 or 0 (for a missing or skipped base, we call it a *hole*). Each row ***m****_i_* corresponds to a SNP fragment *f_i_* and each column corresponds to a SNP site. Since the given SNP fragments may have different lengths, but are generally less than *n*, we also assign value 0 to the uncovered elements in a row.

Let *x,y* ∈ {−1, 1, 0} and define

(1) $$d(x,y)=\{\begin{array}{ll} 1 & \text{if }x\neq 0,y\neq 0\;\text{and }x\neq y,\\ 0 & \text{otherwise}, \end{array}$$

then the distance between two SNP fragments *f_i_* = (*m_i_*_1_, ···, *m_in_*) and *f_k_* = (*m_k_*_1_, ···, *m_kn_*) is defined as *HD*(*f_i_*, *f_k_*) = ∑*_j_*_=1_*^n^d*(*m_ij_, m_kj_*). If *HD*(*f_i_*, *f_k_*) > 0, we say two fragments *f_i_* and *f_k_* are *in conflict*, otherwise we call them *compatible. HD*(*f_i_*, *f_k_*) > 0 indicates that either *f_i_*, *f_k_* are not from the same chromosome copy or there are errors in the data. *HD*(*f_i_*, *f_k_*) is similar to the Hamming distance, i.e., the number of mismatches (conflicts) between two fragments. The distance between a fragment and a haplotype is defined in the similar way.

The MEC problem [Lippert et al. 2002; Wang et al. 2005] is defined as: Given a set of SNP fragments, correct a minimum number of SNP states (−1 into 1 and vice versa), such that the modified SNP fragments can be divided into two disjoint sets of pairwise compatible fragments, and each set determines a haplotype. In order to add the information of genotype into MEC, we propose the following combinatorial optimization model for individual haplotyping:

MCIH (Minimum Conflict Individual Haplotyping): *Given a set of SNP fragments (a SNP matrix* ***M****) from an individual’s DNA and the related genotype* ***g****, reconstruct a pair of haplotypes compatible with g and involving a minimum number of conflicts with the given SNP fragments.*

MCIH is in fact to correct a minimum number of SNP states under the guidance of the genotype information so that the modified SNP fragments can be divided into two disjoint sets of pairwise compatible fragments which determine a pair of haplotypes compatible with the genotype. The computational complexity of the MCIH problem (similar spirit with [Eskin et al. 2004]) is discussed in Appendix A where we prove it to be NP-hard. It indicates that the MCIH problem may have no efficient algorithm for exact solutions.

### II. Model Evaluation

The purpose of presenting the new model is to get a high-quality solution of the haplotype assembly problem. To show that MCIH is a potential alternative, we evaluate the model by studying its exact solutions. A special data structure with Markov property suited for a dynamic programming algorithm is considered as follows.

Let *l_i_* and *r_i_* be the beginning and ending positions of the *i*th SNP fragments *f_i_* on the SNP matrix respectively, 1 ≤ *i* ≤ *m*. For any two fragments *f_i_* and *f_j_*, we assume

(2) $$\text{if }l_{i}\leq l_{j},\;\text{then }r_{i}\leq r_{j}.$$

The rows of the SNP matrix is reordered according to the beginning positions and the ending positions of SNP fragments. For two rows *m_i_* and *m_j_*, *i* < j if and only if *l_i_* < *l_j_*. If *l_i_* = *l_j_* and *r_i_* < *r_j_*, the *i*th row will also be put before the *j*th row. From the proof of the computational complexity in Appendix A we know that the MCIH with this special structure remains NP-hard.

To solve MCIH with this special structure, we give a dynamic programming (DP) algorithm. The input data of the DP algorithm are a genotype ***g*** with length *n* and a SNP matrix of the type (2). The outputs of the DP algorithm are a partition of the SNP fragments and a pair of halotypes (***h***_1_, ***h***_2_) generated from the partition. In fact, the DP algorithm is used to reconstruct one haplotype, say ***h***_1_. Clearly, ***h***_2_ can be obtained immediately from ***g*** and ***h***_1_.

Suppose that ***x***(*j*) = (*x_l_*,···, *x_r_*_*_j_*_) is an assignment to the positions *l_j_*, ···, *r_j_* of ***h***_1_, and ***x̄*** (*j*) is the corresponding assignment to ***h***_2_ at the same positions. If *g_k_* ≠ 0, *x_k_* *=x̄_k_* = *g_k_*/2, otherwise *x_k_* + *x̄_k_* = 0 for *k* = *l_j_*, ···, *r_j_*. Let *f_1_*(*j*, ***x***(*j*)) denote the number of conflicts between the SNP fragment *f_j_* and ***h***_1_ with assignment ***x***(*j*), *f_2_*(*j*, ***x̄***(*j*)) denote the number of conflicts between the SNP fragment *f_j_* and ***h***_2_ with assignment ***x̄***(*j*). Define *f* (*j*, ***x***(*j*)) = min{ *f_1_*(*j*, ***x*** (*j*)), *f_2_*(*j*, ***x̄*** (*j*))} which implies the haplotype that the *j*th fragment belongs to. The main steps of the dynamic programming algorithm are as follows:

#### Step 1

Initialization.

Let *j* = 1. *N*(1, ***x***(1)) = *f* (1, ***x***(1)) = min{ *f*_1_(1, ***x***(1)), *f*_2_ (1, ***x̄*** (1))} for all possible ***x***(1).

#### Step 2

Follow the recurrence formula.

Define *N*(*j*, ***x*** (*j*)) as the minimum associated number of conflicts between the first *j* fragments and haplotypes (***h***_1_, ***h***_2_) at positions 1, 2, ···, *r_j_*, with the assignment ***x*** (*j*).

$$\begin{array}{l} N(j+1,x(j+1))=\underset{\left(b_{l_{j}},\cdots ,b_{{l_{j+1}}^{-1}}\right)}{\text{min}}\\ \left\{h_{1}(j,(b_{l_{j}},\cdots ,b_{{l_{j+1}}^{-1}},x_{l_{j}+1},\cdots ,x_{r_{j}})\right)\\ +f(j+1,x\;(j+1))\}, \end{array}$$

where *b_k_* = *g_k_*/2 for *g_k_* ≠ 0, otherwise *b_k_* ∈{1,−1}, *k* = *l_j_*, ···, *l_j_*_+1_ −1.

#### Step 3

Trace the solution to obtain a pair of haplotypes (***h***_1_, ***h***_2_) and a partition of the SNP fragments.

When all *N*(*j*, ***x***(*j*)) for 1 ≤ *j* ≤ *m* are computed by the recurrence formula, we can find *N*(*m*, ***x***(*m*)) for all ***x*** (*m*) = (*x_lm_*, ···, *x_rm_*). Thus, the solution of the MCIH problem can be obtained by tracing the solution forwardly which leads to a minimal value at each *j* from the following formula:

$$\underset{\left(x_{l_{m}},\cdots ,x_{r_{m}}\right)}{\text{min}}N(m,x(m)).$$

For all *j*, *f*(*j*, ***x***(*j*)) = min{*f*_1_(*j*, ***x***(*j*)), *f*_2_(*j*, ***x̄*** (*j*))} determines a partition of the SNP fragments.

Assume that *L* is the maximum length of SNP fragments. It can be shown that the dynamic programming algorithm solving the special MCIH problem has the complexity *O*(2^2^*^L^m*). Hence, the algorithm is exponential with the maximum length of SNP fragments. However, when the maximum length of SNP fragments is fixed, this algorithm is linear to *m*, i.e., the number of fragments. In real applications, the value of *L* is generally between 3 and 8.

As expected, numerical results (see Appendix B for details) of DP on the special data structure display that MCIH at the cost of genotype information improves the reconstruction rate greatly at various error rates of SNP fragments. An extreme case is considered where every SNP site is heterozygous. It means that the model has no homozygous site information available from the given genotype. The numerical results show that in this case MCIH still has a higher reconstruction rate than MEC. This indicates that MCIH is not trivial, i.e., a higher reconstruction rate does not only depend on homozygous site information. Heterozygous site information has much contribution to the accuracy of the reconstructed haplotypes.

### III. A Feed-Forward Neural Network Algorithm

As discussed in Introduction, the MCIH problem is actually a classification problem. That is, given a set of SNP fragments, we want to classify it into two fragment subsets such that each subset determines a haplotype to solve the problem with minimum conflicts. It is well known that feed-forward neural network (FNN) is a powerful tool for classification. In general, an FNN maps a set of objects into classes *C*_1_, ···, *C_s_* characterized by the attributes of the objects through repeatedly learning the objects and adjusting its parameters (neuron connection weights). In our problem, a set of SNP fragments are to be divided into two classes such that each class can be assembled as one of the haplotypes which will solve the problem with as few conflicts as possible.

**Figure 1:**
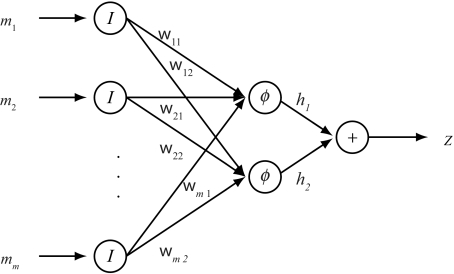
A three layer forward neural network.

The proposed FNN (see Figure 1) consists of three layers. The *m* input neurons represent *m* SNP fragments. Each input neuron accepts an *n*-dimensional vector on {1, −1, 0}. Two hidden neurons represent two subsets of fragments corresponding to one pair of haplotypes. The input dimension of the hidden neuron is also *n* and the outputs of the two neurons are a pair of tentative haplotypes. There is only one neuron in the last layer, which simply conflates the tentative haplotypes and outputs a tentative genotype. Comparing this tentative genotype with the given genotype will provide us the information to adjust the neuron connection weights by the popular back-propagation algorithm (see Appendix B and related literatures for feed-forward neural networks [Rumelhart et al. 1986; Zhang, 2000]). The main characteristic of the designed neural network is trying to achieve two objectives, i.e., compatibility and minimum number of conflicts simultaneously.

#### Forwarding process of FNN

The forwarding process of FNN can be stated as follows.

1. The inputs and outputs of the first layer are *m* rows of the SNP matrix, ***m***_1_, ···, ***m****_m_*, i.e., *m* SNP fragments *f_i_*, *i* = 1, ···, *m*. In fact, the neurons in the first layer are trivial identity maps *I*.
2. There are 2*m* parameters, i.e., the weights from the first layer to the second layer,
  $$W_{m\times 2}=\left(\begin{array}{cc} w_{11} & w_{12}\\ w_{21} & w_{22}\\ \cdots & \cdots \\ w_{m1} & w_{m2} \end{array}\right).$$
3. The inputs to the second layer are ***y***_1_ = (*y*_11_, ···, *y*_1_*_n_*) and ***y***_2_ = (*y*_21_, ···, *y*_2_*_n_*), where
  (3) $$y_{lk}=\sum_{i=1}^{m}m_{ik}\;w_{il},\;l=1,2,\;k=1,\cdots ,n.$$
  Two neurons in the hidden layer have the same sigmoidal neuron function:
  (4) $$\varphi \;(\lambda x)=\text{tanh}\;(\lambda x)=\frac{1-e^{-2\lambda x}}{1+e^{-2\lambda x}}.$$
4. The outputs of the second layer neurons are ***h***_1_ = (*h*_11_, ···, *h*_1_*_n_*) and ***h***_2_ = (*h*_21_, ···, *h*_2_*_n_*), where
  (5) $$h_{lk}=\varphi (\lambda y_{lk}),\;l=1,2,\;k=1,\cdots ,n.$$
5. The inputs to the third layer are ***h***_1_ and ***h***_2_. The neuron function of the third layer is a linear summation, i.e., the output of the third layer is *z* = (*z*_1_, ···, *z_n_*), where
  (6) $$z_{k}=h_{1k}+h_{2k},k=1,\cdots ,n.$$

#### Learning rules

The MCIH problem is to find a pair of haplotypes (***h***_1_, ***h***_2_) to

*Objective-1*: minimize

(7) $$\sum_{f_{i}\in F_{1}}HD\;(h_{1},f_{i})+\sum_{f_{i}\in F_{2}}HD\;(h_{2},f_{i})$$

for all partitions (*F*_1_, *F*_2_) of *f*_1_, ···, *f_m_* and all possible pairs of haplotypes (***h***_1_, ***h***_2_), and

*Objective-2*: satisfy

(8) $$h_{1k}+h_{2k}=g_{k},\;k=1,\;\cdots ,n,$$

or minimize

(9) $$\sum_{k=1}^{n}\left(h_{1k}+h_{2k}-g_{k}\right),\;k=1,\;\cdots ,n,$$

for all possible pairs of haplotypes (***h***_1_, ***h***_2_), where ***g*** = (*g*_1_, *g*_2_, ···, *g_n_*) is the given genotype.

##### 1) Learning to satisfy objective-1

The distance between ***h***_l_ and ***m****_i_* (i.e., *f_i_*) is defined as

$$HD(h_{l},m_{i})=\sum_{k=1}^{n}d(\text{sgn}(h_{lk}),m_{ik}),$$

where *d* is defined by the formula (1) and

$$\text{sgn}(x)=\{\begin{array}{cc} 1, & x\geq 0,\\ -1, & x<0. \end{array}$$

Classify all SNP fragments into two disjoint sets according to their distances to ***h***_1_ and ***h***_2_, i.e.,

(10) $$F_{1}=\{f_{i}:HD(h_{1},m_{i})<HD(h_{2},m_{i}),i=1,\cdots m\},$$

(11) $$F_{2}=\{f_{i}:HD(h_{2},m_{i})\leq HD(h_{1},m_{i}),i=1,\cdots m\}.$$

For the neuron corresponding to ***h***_1_ in the second layer, the network learns to minimize the following error function between ***h***_1_ and the SNP fragments in *F*_1_:

(12) $$R_{11}=\sum_{f_{i}\in F_{1}}\sum_{k=1}^{n}{\left(h_{1k}-m_{ik}\right)}^{2}|m_{ik}|.$$

For the neuron corresponding to ***h***_2_, the network learns to minimize the error function between ***h***_2_ and the SNP fragments in *F*_2_:

(13) $$R_{12}=\sum_{f_{i}\in F_{2}}\sum_{k=1}^{n}{\left(h_{2k}-m_{ik}\right)}^{2}|m_{ik}|.$$

##### 2) Learning to satisfy objective-2

The objective that the third layer adjusts to is to minimize the difference between the tentative genotype ***z*** and the original genotype ***g***:

(14) $$R_{2}=\sum_{k=1}^{n}{\left(z_{k}-g_{k}\right)}^{2}.$$

#### A Back-Propagation-like procedure

The main steps of the algorithm is as follows:

##### Initialization

Set parameter values *L*_1_, *L*_2_, *ρ*, λ and *ɛ*. Randomly set the initial values of the weight matrix ***W***(0) with entries *w_il_* ∈ [0, 1], *i* = 1, ···, *m*, *l* = 1, 2. *t* = 0.

###### Step 1—Feed-forwarding

Input a SNP matrix. Do computation of (3), (5) and (6) to get a pair of tentative haplotyes (***h***_1_, ***h***_2_).

###### Step 2—Learning

*Substep 2.1:* Obtain a fragment partition (*F*_1_, *F*_2_) using (***h***_1_, ***h***_2_) according to the formulae (10) and (11).

*Substep 2.2:* Calculate the gradients of the error functions ∇*_w_*_1_*R*_11_, ∇*_w_*_2_*R*_12_, ∇*_w_*_1_*R*_2_, ∇*_w_*_2_*R*_2_ according to (17), (18), (19), and (20).

*Substep 2.3:* Update the current weight matrix ***W*** (*t*) by

$$\begin{array}{l} w_{1}(t+1)=w_{1}(t)-\rho \Delta w_{1},\\ w_{2}(t+1)=w_{2}(t)-\rho \Delta w_{2}, \end{array}$$

where

(15) $$\Delta w_{1}=L_{1}\nabla_{w_{1}}R_{11}+L_{2}\nabla_{w_{1}}R_{2},$$

(16) $$\Delta w_{2}=L_{1}\nabla_{w_{2}}R_{12}+L_{2}\nabla_{w_{2}}R_{2},$$

*ρ* is the step length along the negative gradient. *L*_1_ and *L*_2_ are parameters to be selected.

*Substep 2.4:* If ||***W*** (*t* + 1) − ***W*** (*t*)|| < *ɛ*, go to Step 3, otherwise return to Step 1.

###### Step 3—Output

Record a pair of haplotypes (***ĥ***_1_*,* ***ĥ***_2_) and a genotype ***ĝ*** as the output.

The formulae to compute the derivatives in the algorithm are given in Appendix B. In contrast to DP algorithm that gives an exact solution, the algorithm based on the neural network can not ensure an exact optimal solution but is proved very efficient for large-scale problems by the numerical results in next section.

### IV. Simulation and Results

In this section, we will use both real data and simulation data to test MCIH and the FNN algorithm for haplotype reconstruction. The computation is implemented on a 2.26G Hz Pentium 4 processor PC using Microsoft Visual C++ compiler 6.

In our experiments, we use *reconstruction rate* (*RR*), the similarity degree between the original haplotypes and the reconstructed haplotypes, to measure performance of an algorithm or a model. Assume that ***h*** = (***h***_1_, ***h***_2_) is the original pair of haplotypes, and ***ĥ*** = (***ĥ***_1_*,* ***ĥ***_2_) is the reconstructed pair of haplotypes. We define *RR* as:

$$RR\;(h,\hat{h})=1-\frac{\text{min}\;\{r_{11}+r_{22},r_{12}+r_{21}\}}{2n},$$

where *r_ij_* = *HD* (*h_i_, ĥ_j_*), *i* = 1, 2, *j* = 1, 2. We use *compatibility rate* (*CR*) to measure the similarity degree between the original genotype ***g*** and the reconstructed genotype ***ĝ***:

$$CR\;(g,\hat{g})=\frac{HD\;(g,\hat{g})}{n}.$$

For a partition *P* = (*F*_1_, *F*_2_) and a pair of haplotypes (***h***_1_, ***h***_2_), the corresponding conflict number is defined by (7).

In this paper, we set step length *ρ* = 0.05, *ɛ* = 0.01 and λ = 0.1 (in fact, the algorithm is robust with these parameters) in the algorithm. In (15) and (16), we set *L*_1_ = 0.2 and *L*_2_ = 0.8.

#### Experiment on angiotensin-converting enzyme (ACE)

Angiotensin-converting enzyme catalyses the conversion of angiotensin I to the physiologically active peptide angiotensin II, which controls fluid-electrolyte balance and systemic blood pressure. Because it has a key function in the renin-angiotensin system, many association studies have been performed with DCP1 (encode angiotensin-converting enzyme). Literature [Rieder et al. 1999] completed the genomic sequencing of the DCP1 gene from 11 individuals and reported 78 SNP sites in 22 chromosomes. Out of the 78 varying sites, 52 are non-unique polymorphic sites.

Among these 11 individuals, there are two identical genotypes. We omit one of them. In addition, we omit the genotypes with no more than one heterozygous site whose haplotypes can be infered immediately. Now each of the 8 pairs of haplotypes is used to generate 15 instances in which SNP fragments are randomly generated according to different parameter settings: the number of SNP fragments *m* = 20; other parameters, the *hole rate* of fragments *hr:* 0.25, 0.5, 0.75; the *error rate* of fragments *e*: 0.05, 0.1, 0.15, 0.2 and 0.25.

**Figure 2:**
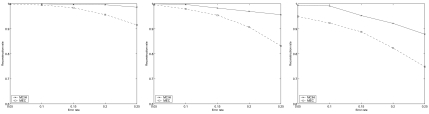
The results of MCIH and MEC on ACE. From left to right, *hr* = 0.25, *hr* = 0.5, *hr* = 0.75.

The results of MCIH and MEC (solved by an exact algorithm in [Wang et al. 2005]) averaged on eight individuals is illustrated in Figure 2. All the results are obtained by running the algorithms only once. When the error rate of SNP fragments is low, the neural network is robust and efficient. When the error rate of SNP fragment is high, the network may get into a plight of local minima. The genotype compatibility rate returned by the algorithm for most instances is 100% or at least larger than 98%. Only several instances with error rate 0.25 and hole rate 0.75 have genotype compatibility rate between 94% and 96%. In addition, the neural network algorithm solves each of these instances in several seconds.

Figure 2 shows that MCIH has a much higher reconstruction rate at various parameter settings, which indicates that with additive genotype information MCIH is effective. Even if an approximate algorithm is employed, it is more effective for haplotype reconstruction than MEC.

#### Experiment on data from chromosome 5*q*31

Now we performed simulations using the data from public Daly set [Daly et al. 2001]. They reported a high-resolution analysis of a haplotype structure across 500kb on chromosome 5*q*31 using 103 SNPs in a European derived population which consists of 129 trios. The haplotypes of 129 children from the trios can be infered from the genotypes of their parents through pedigree information and the nontransmitted chromosomes as an extra 129 (pseudo) haplotype pairs. Markers for which both alleles could not be inferred are marked as missing. Among the resulting set of 258 haplotype pairs, the ones with more than 20% missing alleles are removed, which leaves us 147 haplotype pairs. Among these pairs, 18 genotypes with no more than one heterozygous site are omitted, then 129 pairs of haplotypes are left as the test set.

**Figure 3:**
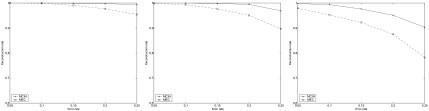
The results of MCIH and MEC on Daly set. From left to right, *hr* = 0.25, *hr* = 0.5, *hr* = 0.75.

Each of the 129 pairs of haplotypes is used to generate 15 instances in which SNP fragments are randomly generated according to different parameter settings: *m* = 30; other parameters, *hr*: 0.25, 0.5, 0.75; *e*: 0.05, 0.1, 0.15, 0.2 and 0.25. The results of MCIH and MEC averaged on 129 pairs of haplotypes are illustrated in Figure 3.

The picture again shows that MCIH is quite effective and the designed back-propagation-like algorithm has a very good performance. The genotype compatibility rate returned by the algorithm for most instances is 100% or greater than 98%. Only a few instances with error rate 0.25 and hole rate 0.75 have genotype compatibility rate between 92% and 96%. In addition, the neural network algorithm solves instances with a low error rate and hole rate in several seconds. For a few instances with a high error rate and hole rate, however, it takes the algorithm several minutes to stop.

#### Experiments on Hudson’s simulation data

**Figure 4:**
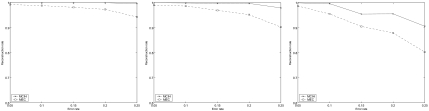
The results of MCIH and MEC on Hudson’s data with *r* = 0. From left to right, *hr* = 0.25, *hr* = 0.5, *hr* = 0.75.

**Figure 5:**
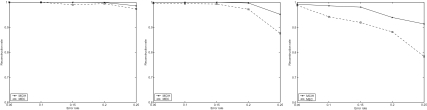
The results of MCIH and MEC on Hudson’s data with *r* = 100. From left to right, *hr* = 0.25, *hr* = 0.5, *hr* = 0.75.

We use a well-known program *ms* [Hudson, 2002] that uses coalescent theory to generate a simulated population of haplotypes. *ms* has a parameter *r* as the recombination rate of population haplotypes. Firstly, let *r* = 0, then 20 haplotypes with 80 SNP sites are generated using *ms* and form a haplotype set. Then we randomly combine two halotypes in the haplotype set into a pair of individual haplotypes. 15 pairs of haplotypes are obtained by this way. Each of the 15 pairs of haplotypes is used to generate 15 instances according to the parameters as those in the last subsection. All of these instances form a dataset. The results of the two models averaged on 15 pairs of haplotypes are shown in Figure 4. To further evaluate MCIH, let *r* = 100 and other parameters be the same. The results of two models on this dataset are summarized in Figure 5. The genotype compatibility rate for almost all instances is 100% or larger than 98%.

From Figure 4 and Figure 5 we can see that MCIH is effective on haplotypes not only without recombination but also with a high recombination rate.

## V. Conclusion

Individual haplotyping is an important problem in computational biology. In this paper, we proposed a new computational model for individual haplotyping—MCIH as an improvement of MEC and as an alternative way for biologists to solve the haplotyping problem more efficiently. The model is proved to be NP-hard. To evaluate the new model, we displayed a special SNP matrix structure for which a dynamic programming algorithm can be used to solve MCIH exactly. Comparing the exact solutions of MCIH and MEC, we argue that the proposed MCIH is worth further studying. Due to the computational intractability of the MCIH problem, a feed-forward neural network is designed and a back-propagation-like procedure is formed as an efficient approximate algorithm. Computational results on multiple data sets show that the designed algorithm performs well and MCIH at the cost of additive genotype information has a higher accuracy of haplotype reconstruction than MEC, especially in the case of SNP fragments with a high error rate. Since genotype information can be obtained much easily and economically, the new model is practical as a supporting tool for reconstruction of haplotypes.

## Figures and Tables

**Table 1:** The results of two models on simulated data.

error rate	s=0.5		s=0.0	
	
	MEC	MCIH	MEC	MCIH
0.05	0.941	1.000	0.965	0.996
0.1	0.904	0.969	0.950	0.984
0.15	0.863	0.969	0.890	0.946
0.2	0.786	0.908	0.834	0.922
0.25	0.763	0.863	0.766	0.830

## Appendix

### A. The MCIH problem is NP-hard

#### Proof

The NP-hardness of the MCIH problem can be obtained by reduction from MAX-CUT. Let *G* = (*V,E*) be an instance of MAX-CUT with a cut of at least size *r*. Then an instance of the MCIH problem is constructed as follows: Define |E| SNP fragments with all of the SNP fragments having length of |*V*|+|*E*|. The genotype ***g*** has length of |*V*|+ 2|*E*| − 1. Let {|*E*|+1, |*E*|+2, · · ·, |*E*|+|*V*|} be the set of positions on the genotype that corresponds to vertices of *G*. Define the genotype at these positions as ambiguous sites, i.e., the genotype has values of 0 at these positions. Let other positions be homozygous sites having a value of 2. Each edge *e* corresponds to a SNP fragment *f**_e_*. For convenience, we label the edges as *e*_1_, *e*_2_, …, *e*_|_*_E_*_|_. The SNP fragment *f**_e_*_*_k_*_ indicated by the edge *e**_k_* begins at the *k*th position of the genotype. Note that all SNP fragments share the positions indicated by vertices of *G*. If *e**_k_* = (*v**_i_**, v**_j_*), then we define (*f**_e_*_*_k_*_ (*v**_i_*) = 1, *f**_e_*_*_k_*_ (*v**_j_*)= −1. For other vertex positions or positions that *f**_e_*_*_k_*_ do not cover, we let *f**_e_*_*_k_*_ (*v*) = 0, i.e., a hole site. For each non-vertex position *l* covered by, *f**_e_*_*_k_*_, *f**_e_*_*_k_*_ (*l*) = 1.

We now prove that *G* has a cut of at least size *r* if and only if the above MCIH instance has a solution with at most |*E*| − *r* conflicts. Firstly, assume that the above MCIH instance has a solution with at most |*E*| − *r* conflicts. Let (***h***_1_*,* ***h***_2_) be a pair of haplotypes which is compatible with the genotype ***g*** and has minimum number of conflicts with the given SNP fragments. We know ***h***_1_(*v**_i_*) ≠ ***h***_2_(*v**_i_*) for each vertex position *v**_i_*. Let *V*_1_ = {*v**_i_* ∈ *V* | ***h***_1_(*v**_i_*) =1} and *s* be the number of edges crossing the cut created by *V*_1_. For each edge *e**_k_* = (*v**_i_**, v**_j_*), if ***h***_1_(*v**_i_*) ≠ ***h***_1_(*v**_j_*), then the number of conflicts between (***h***_1_, ***h***_2_) and *f**_e_*_*_k_*_ is zero. If ***h***_1_(*v**_i_*) = ***h***_1_(*v**_j_*), the number of conflicts is 1. So the total number of conflicts is:

$$\begin{array}{l} \left|\{(v_{i},v_{j})\in E|h_{1}(v_{i})=h_{1}(v_{j})\}\right|\\ =(|E|-|\{(v_{i},v_{j})\in E|h_{1}(v_{i})\neq h_{1}(v_{j})\}|\\ =|E|-s\leq |E|-r. \end{array}$$

Hence *s* ≥ *r*, i.e., *G* has a cut with size at least *r*. On the other hand, if (*V*_1_, *V̄*_1_) be a cut of *G* of at least size *r*, then we define ***h***_1_ to have value of 1 at non-vertex positions. For a vertex position *v**_i_*, if *v**_i_* ∈ *V*_1_, then ***h***_1_(*v**_i_*) = 1, else ***h***_1_(*v**_i_*) = −1. ***h***_2_ can be obtained from ***g*** and ***h***_1_. We can see that such a pair of haplotypes is compatible with ***g*** and has at most |*E*| − *r* conflicts with the SNP fragments.

### B. Evaluation MCIH on special SNP matrix—DP algorithm

In this part, we use simulation data to evaluate MCIH with an exact algorithm—dynamic programming algorithm. 100 pairs of haplotypes with 25 SNP sites are randomly generated according to a parameter *similarity rate s* (the similarity degree between two haplotypes in one pair). Then, each of 100 pairs of haplotypes is used to generate 5 instances in which SNP fragments are randomly generated according to *error rate e*: 0.05, 0.1, 0.15, 0.2 and 0.25. The lengths of SNP fragments are between 6 and 8. The number of SNP fragments in each instance is between 20 and 40. We first let *s* = 0.5. The results of MCIH and MEC (solved by an exact algorithm in [Wang et al. 2005]) averaged on 100 pairs of haplotypes are summarized in Table 1. The dynamic programming algorithm solves each instance in no more than one second.

As expected, MCIH, which employs genotype information, raises the reconstruction rate greatly at various error rates of SNP fragments. In order to evaluate MCIH more objectively, we let *s* = 0, i.e., every SNP site in the genotypes corresponding to 100 pairs of haplotypes is a heterozygous site (this is an extreme case). Table 1 shows that in this case MCIH has a higher reconstruction rate than MEC even without employing homozygous site information. This indicates that MCIH is not trivial, i.e., a higher reconstruction rate does not only depend on homozygous site information. Heterozygous site information also has much contribution to the accuracy of the reconstructed haplotypes.

### C. Calculation of the gradients of error functions

Since **φ̇** (*λx*) = *λ*(1 − *φ*^2^(λ*x*)), the gradient of *R*_11_ at ***w***_1_ = (*w*_11_, *w*_21_, ···, *w**_m_*_1_)*^T^* consists of

(17) $$\frac{\partial R_{11}}{\partial w_{i1}}=\{\begin{array}{ll} \sum_{k=1}^{n}2[\varphi \;(\lambda y_{1k})-m_{ik}]\cdot \lambda \;[1-\varphi^{2}(\lambda y_{1k})]\;m_{ik} & \text{for }f_{i}\in F_{1}\\ 0 & \text{for }f_{i}\notin F_{1} \end{array}$$

where *i* = 1, 2, ···, *m*. The gradient of *R*_12_ at ***w***_2_ = (*w*_12_, *w*_22_, …, *w**_m_*_2_)*^T^* has

(18) $$\frac{\partial R_{12}}{\partial w_{i2}}=\{\begin{array}{ll} \sum_{k=1}^{n}2[\varphi \;(\lambda y_{2k})-m_{ik}]\cdot \lambda \;[1-\varphi^{2}(\lambda y_{2k})]\;m_{ik} & \text{for }f_{i}\in F_{2}\\ 0 & \text{for }f_{i}\notin F_{2} \end{array}$$

where *i* = 1, 2, ···, *m*.

Similarly, we have

(19) $$\frac{\partial R_{2}}{\partial w_{i1}}=\sum_{k=1}^{n}2[\varphi \;(\lambda y_{1k})+\varphi \;(\lambda y_{2k})-g_{k}]\cdot \lambda \;(1-\varphi^{2}(\lambda y_{1k}))\;m_{ik},$$

and

(20) $$\frac{\partial R_{2}}{\partial w_{i2}}=\sum_{k=1}^{n}2\;[\varphi \;(\lambda y_{1k})+\varphi \;(\lambda y_{2k})-g_{k}]\cdot \lambda \;(1-\varphi^{2}(\lambda y_{2k}))\;m_{ik},$$

where *i* = 1, 2, ···, *m*.
